# Quality of top webpages providing abortion pill information for Google searches in the USA: An evidence-based webpage quality assessment

**DOI:** 10.1371/journal.pone.0240664

**Published:** 2021-01-21

**Authors:** Elizabeth Pleasants, Sylvia Guendelman, Karen Weidert, Ndola Prata

**Affiliations:** 1 Wallace Center for Maternal, Child, and Adolescent Health Research, School of Public Health, University of California, Berkeley, Berkeley, California, United States of America; 2 School of Public Health, University of California, Berkeley, Berkeley, California, United States of America; 3 Bixby Center for Population, Health and Sustainability, University of California, Berkeley, Berkeley, California, United States of America; Georgia Southern University, UNITED STATES

## Abstract

**Background:**

In the United States, the internet is widely used to seek health information. Despite an estimated 18 million Google searches on abortion per year and the demonstrated importance of the abortion pill as an option for pregnancy termination, the top webpage search results for abortion pill searches, as well as the content and quality of those webpages, are not well understood.

**Methods:**

We used Google’s Custom Search Application Programming Interface (API) to identify the top 10 webpages presented for “abortion pill” searches on August 06, 2018. We developed a comprehensive, evidence-based Family Planning Webpage Quality Assessment Tool (FPWQAT), which was used to assess webpage quality for the five top webpages presenting text-based educational content.

**Results:**

Of the top webpages for “abortion pill” searches, a plannedparenthood.com page was the top result and scored highest on our assessment (81%), providing high-quality and useable information. The other four webpages, a Wikipedia.com page and three anti-abortion information webpages, scored much lower on our assessment (14%-43%). These four webpages had lower quality of information in less useable formats. The anti-abortion pages also presented a variety of disinformation about the abortion pill.

**Conclusions:**

Both the lack of accurate clinical content on the majority of top webpages and the concerning disinformation they contained raise concerns about the quality of online abortion pill information, while underlining challenges posed by Google search results to informed choice for consumers. Healthcare providers and consumers must be informed of online abortion pill content that is not based in current clinical evidence, while advocates and policymakers should push for online information that is credible and useable. These changes are imperative given the importance of sound abortion pill information for reproductive decision-making at a time when in-person abortion services are further challenged in the US.

## Introduction

Twenty-nine million women of reproductive age in the United States (US) live in states considered very hostile or hostile to abortion (43% of women of reproductive age as of 2018). For these women, abortion care access is severely restricted by cutbacks in services, legal restrictions that inhibit access, abortion stigma, and fear of potential legal repercussions if a pregnancy were to be terminated [1]. It has also been found that in more restrictive states, simulated patient callers (representing abortion seekers) are less likely to receive a referral to an abortion provider and that women are more likely to perceive abortion access as difficult [2, 3]. Additionally, research has shown that women who have to travel long distances to get abortion care may face notable information-related barriers to access [4]. As a result, many individuals face real and perceived difficulties that shape their trajectory of seeking and potentially accessing abortion as a desired healthcare service. Challenges to abortion access along with increases in use of the internet over the past 20 years, including its use as a resource for health information and services, could lead to growing reliance upon the internet to search for information and to locate abortion care resources [1, 4–7].

The majority of people who search for information online use the Google search engine, making it a key source of information [8]. We previously estimated that there were between 16.4 and 18.9 million Google searches on abortion in the US in 2017 [9]. Guendelman et al. examined the most highly searched queries in connection with abortion in the US in 2018 and found a high frequency of information seeking for “abortion pill” [10]. The top search queries—ranked by the strength of its association with abortion—were abortion pill, abortion pill cost, abortion clinic(s), Planned Parenthood, abortion facts, abortion statistics, and partial birth abortion. In terms of the quality of abortion webpages, previous studies have looked at the overall quality of Google search results, highlighting the barriers to abortion access they pose. A study of top search results for abortion seekers in 68 cities by Dodge et al. found that only 53% of webpage results facilitated abortion self-referrals while 13% obstructed self-referral. Anti-abortion pages such as crisis pregnancy centers (CPCs) occurred more frequently when the nearest abortion clinic was at least 100 miles away from the location connected to the search [11]. The presence of disinformation on anti-abortion pages, such as those more commonly presented to users in low access-areas in Dodge et al.’s study, has been well demonstrated and explored as a challenge to abortion access [12–14].

The “abortion pill” or medication abortion (hereafter referred to as the “abortion pill”) generally refers to the use of mifepristone followed by misoprostol for pregnancy termination. This regimen for pregnancy termination was approved for use in the United States in 2000, after 12 years of political and social conflict following its discovery [15]. The American College of Obstetricians and Gynecologists (ACOG) currently endorses the abortion pill as a safe and highly effective method for pregnancy termination up to 70 days after the patient’s last menstrual period [16–19]. Out of all induced abortions in the US, abortion pill use accounted for just 6% in 2001 and almost 40% in 2017 [20]. Steadily increasing abortion pill use has been paralleled by growing evidence of its safety and efforts to reduce access barriers across the US, as with allowances for administration via telemedicine and by mid-level healthcare providers [20–22]. Nonetheless, access to the abortion pill has been systematically challenged by anti-abortion legislation introduced in both the US House and Senate, as well as a variety of state-level provisions, aimed at stigmatizing the procedure while promoting and legitimizing abortion pill disinformation [20, 22].

We aimed to: (1) identify the top webpages providing information to people searching for “abortion pill” on Google within the US and; (2) ascertain the extent to which those webpages are “high-quality” based upon assessment using a Family Planning Webpage Quality Assessment Tool (FPQWAT) developed for this project. This tool incorporated evidence on effective communication and optimization of user experience of health information [23–34], and clinical guidelines and factual information for abortion pill counseling and care [35]. Webpage quality refers to the extent to which a webpage provides comprehensive, evidence-based, and usable information (useable information is defined as information that can be read, understood, and acted upon by the user). By identifying the top webpages for abortion pill searches and assessing the quality of each webpage, our analysis provides a better understanding of the landscape of online abortion pill information, including challenges that online information seekers face, while facilitating the promotion of high-quality webpages as resources for consumers.

## Materials and methods

### Top webpages methodology

Past studies have used Google Trends to determine what information people search for on Google in “real” time. While the publicly available Google Trends interface is a popular and user-friendly tool, it relays only information relating to search queries (what users searched for), not search results [36]. Notably, for each search query on Google, the first page of webpage results lists approximately ten webpages determined by their search engine optimization algorithm [37]. These pages receive the majority of clicks from searchers. As found in Chitika’s 2013 study of 300 million webpage visits in the US and Canada, the “webpage with the first position in the search results contributed to 33% of the traffic, compared to 18% for the second position;” the first ten pages in the search results received at least 92% of the traffic [38].

A protocol was recently developed to connect the most popular search terms with the top webpages in the contiguous US using Google’s Custom Search API [39]. This methodology effectively links search queries to a ranked list of the top 10 webpage results at the time of the API query. We applied this protocol to identify the top 10 webpages for abortion pill searches on Google within the contiguous US as of August 06, 2018. Based on click-through estimations, the ranked list of top 10 webpages for abortion pill searches resulting from our methodology would characterize the webpages receiving the majority of search traffic for abortion pill searches in the US.

### Webpage quality assessment methodology

#### Tool development

Our team developed the FPWQAT to assess the quality of webpages for a range of family planning methods, accounting for the specific information needed to assess clinical and factual accuracy for each method by creating method-specific sections within the FPWQAT. As part of a larger study, we applied the FPWQAT to top webpages providing educational content for a variety of methods. In this analysis we used the same methodology to assess the quality of top webpages for abortion pill searches. The FPWQAT was developed using the following steps:

*Literature search for assessments of the quality of webpages and online health information*. We compiled and synthesized peer-reviewed articles describing previous quality assessments of online sexual and reproductive health information, as well as those describing assessments of user experience of webpages presenting health information published since 2000 (with a few exceptions for foundational publications). We examined 28 scholarly articles on these topics pulled from PubMed and Google Scholar and extracted two main findings. First, certain webpage characteristics such as clearly stated page objectives, easy to use top navigation, the presence of an internal search engine on the page, and use of language at a reading level most users can comprehend, lend a page to be more “user-friendly” and to communicate content more effectively [24–34]. Second, while tools have been developed to assess the quality of online sexual and reproductive health information for specific contraceptive methods (IUD [40] and oral contraceptives [41]) or age groups [42], no comprehensive tool existed to assess information across methods of contraception and abortion, or to assess webpages providing information on the abortion pill specifically.

*Review and synthesis of clinical and factual information*. We conducted an additional search with the goal of gathering all of the content needed to assess the quality of clinical and factual information presented by webpages on the abortion pill. Resources included documents from ACOG, the Association of Reproductive Health Professionals, and the Centers for Disease Control (CDC) [17, 18, 35, 43]. Information from these resources was synthesized into a list of the assessment criteria necessary to determine if a page could provide a consumer with comprehensive and useable information on the abortion pill.

*Initial quality assessment tool development*. Informed by the literature, we developed an initial draft of the FPWQAT with 128 criteria to assess method-specific clinical and factual information as well as the relevance, timeliness, attractiveness, usability, and accuracy of webpages as resources for textual content for health education.

*Narrowing assessment criteria*. The entire research team reviewed the initial tool and refined it based on relevance and utility to this analysis, eliminating criteria that did not yield meaningful or valid information for our assessment. For example, because all pages met the criterion for few spelling or grammar errors the assessment criterium was removed from the FPWQAT. Additionally, if a criterion could not be applied consistently even after discussions to attempt to reach a consensus within the research team, it was modified to facilitate greater uniformity in assessment.

*Tool pre-testing*. The assessment criteria in the FPWQAT were then applied by three members of the team (EP, KW, NP) to a small subsample of webpages to test the relevance, clarity, and objectivity of assessment criteria based on application. For our assessment, we defined a “webpage” as all content contained within the linked URL; if there were multiple pages on a website covering the same content area that a user would click through, as with forward and back arrows built into the educational content, all pages were assessed as one “webpage.” Criteria wording was further refined based on pre-testing. Assessments done during pre-testing were not included in final results.

*Expert feedback*. We then sought input from six experts to further refine the FPWQAT. Four experts in marketing, health communications, and health informatics were purposively sampled from the Bay Area; these experts had at least five (between 6 and 15) years of experience in their respective fields and each demonstrated expertise in the design and/or evaluation of online content with a focus on effective communication of information to consumers. We also consulted two additional experts with publications demonstrating experience in abortion, contraception, and online information within the US. All experts reviewed the FPWQAT and provided written or verbal feedback on the tool. Based upon expert input, the tool was revised to include 116 clearly described assessment criteria to effectively assess the quality of webpages providing textual family planning education to the public, including those specific to the abortion pill. During this refinement, the study team discussed the effective application of assessment criteria, continuing to develop a strong internal consensus on the use of the FPWQAT.

*Final tool*. The final product was the FPWQAT with two sections, one to assess Quality of Information (QI) on the webpage specific to each family planning method and another to assess the User Experience (UX) of the webpage (see the complete FPWQAT in S1 Appendix). When the FPWQAT was applied to abortion pill webpages, the first section evaluated the QI on the webpage, designed to assess the presence and accuracy of clinical information and facts pertinent to abortion pill access and uptake in the US. Scoring for this section is broken down into two sub-scores—one for clinical information and one for abortion pill related facts. Scores reflect the extent to which pages presented accurate clinical information, such as abortion pill side effects and contraindications, separately from how they presented factual information, such as abortion pill cost and potential barriers to access like abortion restrictions in the US. For QI assessment criteria, information had to be present and accurate on the webpage to be scored as a “Yes” (1); if information was absent or inaccurate based on our criteria it was scored as “No” (0). Summary statements for each of the 10 abortion pill assessment criteria are presented in **Table 1.**

**Table 1:** Quality of information assessment criteria for abortion.

Abortion Pill: Quality of Information Assessment Criteria
***Clinical information*:**
1. Webpage correctly identifies all of the contraindications for the abortion pill as outlined by ACOG [35]
2. Webpage correctly describes the procedure for receiving the abortion pill [35]
3. Webpage correctly describes the usual patient experience during use of the abortion pill to terminate a pregnancy [35]
4. Webpage correctly describes possible complications of the abortion pill and low risk posed by the procedure [35]
5. Webpage correctly describes efficacy of the abortion pill [35]
6. Webpage clearly states that the abortion pills have no long-term health effects [35]
***Facts*:**
7. Webpage makes some reference to how common abortion is in the US [44]
8. Webpage correctly describes the cost of the abortion pill, reflecting cost within the described range [45]
9. Webpage explains that some states have laws that restrict and regulate abortion, does not have to explain in detail but should mention these state laws and their potential impacts on abortion access [46]
10. Webpage does not make any incorrect claims or present any disinformation about the abortion pill based on [24, 26, 29] clinical evidence and current best practice for abortion providers [35]
User Experience Assessment Criteria
1. Webpage does not have advertisements [27]
2. Organization's objectives are presented. Organization mission and objectives clearly presented somewhere on the webpage being reviewed [23]
3. Webpage content is focused on the application of knowledge by users to lead to their desired behavior/outcomes
4. Webpage has at least one visual element (video or multimedia) that conveys or supports the main message of the page [24, 25, 27, 28]
5. Webpage top navigation is easy to understand and use [24, 25, 29–31]
6. Information about the Googled topic can be easily found on the webpage with clear wayfinding (in headings, subheadings, etc.) [29–32, 34]
7. There is an internal search engine present [24, 25, 42]
8. There is a help or chat function present on the webpage [23]
9. Webpage is written at 6–7 grade reading level [33]
10. Vocabulary is user friendly or well explained [28–32]
11. Webpage is mobile-friendly [47]

The second section of the tool evaluates the *UX* of the webpage and consists of 11 criteria (see **Table 1**). These criteria were designed to assess page credibility, design and functionality, and readability and comprehensibility of information. One assessment parameter included in this section of the tool was whether the page content met the National Institutes of Health (NIH) guidelines that health materials be written at the 6–7 grade reading level required to be understandable to the general public [33]. Reading level was determined based upon a readability consensus score for excerpted text; text was run through a readability checker that assigned a grade reading level to the text based on 8 readability formulas (http://www.readabilityformulas.com/free-readability-formula-tests.php). Knowing that many of these pages would have to use some medical terminology or jargon language, we also assessed if they provided clarification or further information on jargon or medical terminology that users might not be familiar with. UX criteria were scored as a “Yes” if the page had the functionality or met the criteria in the parameter, or as “No” if it did not.

#### FPWQAT application to top webpages

We programmed the complete FPWQAT into SurveyGizmo to facilitate easy data entry over the large number of top webpages for all family planning methods assessed as a part of the larger analysis. We excluded top webpage links on YouTube as we were interested in capturing the quality of webpages dedicated to providing textual education on the abortion pill. While videos often provide educational content, quality measures are not well established [48]; and may not be comparable to that of textual content. Although informative, news articles were also excluded as they were not dedicated educational resources. Top webpages that were sub-pages on the same parent site were collapsed into one result, as they were not unique pages and therefore were already accounted for within the previous top webpage result.

The FPWQAT was completed for each top webpage to reflect whether each criterion was met. While the FPWQAT contained 116 criteria to allow assessment for a range of family planning methods, only 21 of these criteria were applicable, resulting in a score out of 21 possible points for all abortion pill webpages. Two raters scored all pages; ongoing discussions allowed for consistency in assessment approaches. All differences in scoring were noted and discussed until both raters reached a consensus on the final score. Across the abortion pill top pages assessed, raters differed by an average of 1.4 criteria out of 21 per page (7%). This research was exempted from review by the University of California, Berkeley Center for Protection of Human Subjects as the authors could not ascertain the identities of the individuals conducting the internet searches.

## Results

The ranked 10 top webpages for “abortion pill” searches in the US are shown in **Table 2**. Of these top 10 pages, five were omitted from our assessment of webpage quality based on our exclusion criteria.

**Table 2:** Top 10 webpages for “abortion pill” searches across the US on 06 August 2018.

Top page ranking	Top page url
1.	https://www.plannedparenthood.org/learn/abortion/the-abortion-pill
2.	http://americanpregnancy.org/unplanned-pregnancy/abortion-pill/
3.	https://www.plannedparenthood.org/learn/abortion/the-abortion-pill/what-can-i-expect-if-i-take-abortion-pill^1^
4.	http://www.abortionpillreversal.com/
5.	https://www.youtube.com/watch?v=Qx-1VsTrFlk^2^
6.	https://www.smithsonianmag.com/health-medicine/science-behind-abortion-pill-180963762/^3^
7.	https://www.bbc.com/news/world-44089526 ^3^
8.	https://en.wikipedia.org/wiki/Medical_abortion
9.	https://www.abortionprocedures.com/abortion-pill/
10.	https://www.youtube.com/watch?v=lRDnVSMr5j0 ^2^

^1^Page was excluded from quality assessment as it was a secondary page of the same plannedparenthood.com page as page 1.

^2^Page was excluded from quality assessment as the only educational/information content it contained was within a video.

^3^ Page was excluded from analysis as it was a news article, not providing educational/information content.

**Table 3** presents results of FPWQAT assessments of top pages; for criteria-specific scoring results see S1 Table. All page rankings correspond to the webpage’s ranking within the list of top 10 results. All scoring results reflect consensus scores agreed upon by the two raters following individual assessments of each page. Of the five top webpages assessed for quality, two were health services webpages focused on directing patients to service providers associated with the page in addition to providing health information. The plannedparenthood.com page links to Planned Parenthood centers and service providers while the abortionpillreversal.com page directed people who are seeking “immediate help or would like to attempt to reverse the abortion pill” to contact their helpline for connection to local assistance. Two webpages were health education pages (providing health information only) and one was a wikipedia.com page, which is a non-profit open source website with information on all topics. None of these top webpages were government pages.

**Table 3:** Quality scores by assessment area for top 5 abortion webpages for search term “abortion pill” in the US.

Page rank^1^	Webpage	Type of Webpage	*Sub-Score*: *Clinical Information*	*Sub-Score*: *Facts*	Quality of Information (QI) Score	User Experience (UX) Score	Total Score
			*score out of 6 (percent)*	*score out of 4 (percent)*	score out of 10 (percent)	score out of 11 (percent)	score out of 21 (percent)
1	https://www.plannedparenthood.org/learn/abortion/the-abortion-pill	Health services page	*5 (83*.*3%)*	*3 (75*.*0%)*	8 (80.0%)	9 (81.8%)	**17 (81.0%)**
2	http://americanpregnancy.org/unplanned-pregnancy/abortion-pill/ ^2^	Health education page	*1 (16*.*7%)*	*2 (50*.*0%)*	3 (30.0%)	6 (54.5%)	**10 (42.9%)**
4	http://www.abortionpillreversal.com/ ^2^	Health services page	*0 (—)*	*0 (—)*	0 (—)	3 (27.3%)	**4 (19.0%)**
8	https://en.wikipedia.org/wik5i/Medical_abortion	Non-profit (open source information) page	*1 (16*.*7%)*	*2 (50*.*0%)*	3 (30.0%)	6 (54.5%)	**10 (42.9%)**
9	https://www.abortionprocedures.com/abortion-pill/ ^2^	Health education page	*0 (—)*	*0 (—)*	0 (—)	4 (36.4%)	**4 (19.0%)**

^1^Page rank out of pages presenting educational/information content to users, excluding pages on YouTube and news articles, and pages presenting a sub-page of a page already assessed.

^2^ Webpages that are anti-abortion, funded by organizations opposed to abortion and seeking to discourage abortion access.

The plannedparenthood.com page (ranked No. 1) scored higher than all other webpages (81%), with superior scores for QI (80.0%) and UX (81.8%). Compared to the webpages ranked 1 and 4, other top pages presented information at higher reading levels on average (10^th^ grade vs. college level, results not presented here), though none of the pages assessed met the NIH recommendation to present information at a 6–7 grade reading level. Pages ranked 2,4 and 9 also did not explain or define jargon and clinical terminology while pages 1 and 8 did, generally through links to other resources on their webpage. So, in addition to presenting information that was harder to read, they also did not provide any avenue for users to get clarification or definition. Regarding information on method cost, only the pages ranked 1, 2, and 8 provided accurate information on the cost of the abortion pill. We also included a parameter assessing if pages inform users that some states have laws that restrict and regulate abortion; only pages ranked 1 and 2 did this.

The most notable difference across webpages was in the presentation of clinical information. The page on plannedparenthood.com scored far higher than all other pages for QI (83.3%), providing the most accurate and comprehensive clinical information. In QI, this page only lacked correct identification of all contraindications for abortion pill use and any discussion of how common abortion is in the US. Pages ranked 2, 8 and 9 had notably lower scores for QI, with two of the three presenting no correct clinical or factual information at all (pages ranked 4 and 9). These pages also scored poorly for UX, with scores ranging from 27.3 to 54.5%. All three of these sites could be categorized as anti-abortion pages, seeking to deter users from accessing abortion services.

Beyond not providing accurate information about the abortion pill to searchers, the anti-abortion pages assessed presented clinically inaccurate and potentially misleading information related to the effects and use of the abortion pill [36]. The abortion pill disinformation on all top pages is compiled in **Table 4**. Within the three anti-abortion webpages, it was stated that the abortion pill leads to increased risk of mortality (page ranked 2), may have effects on future fertility (page ranked 8), is not appropriate for women with mental health problems (page ranked 2) or that it can lead to mental health problems later (pages ranked 4 and 8), and that the abortion pill is reversible (pages ranked 4 and 9).

**Table 4:** Disinformation provided by commonly returned webpages for abortion pill searches.

Page rank^1^	Webpage	Abortion pill leads to increased risk of mortality	Abortion pill may negative impact future fertility	Abortion pill should not be used by women with mental health issues	Abortion pill can lead to future mental health problems	Abortion pill is reversible	Total
1	https://www.plannedparenthood.org/learn/abortion/the-abortion-pill						0
2	http://americanpregnancy.org/unplanned-pregnancy/abortion-pill/ ^2^	X	X	X	X		4
4	http://www.abortionpillreversal.com/ ^2^				X	X	2
8	https://en.wikipedia.org/wiki/Medical_abortion						0
9	https://www.abortionprocedures.com/abortion-pill/ ^2^				X	X	2

^1^Page rank out of pages presenting educational/information content to users, excluding pages on YouTube and news articles, and pages presenting a sub-page of a page already assessed.

^2^ Webpages that are anti-abortion, funded by organizations opposed to abortion and seeking to discourage abortion access.

## Discussion

This research sought to identify the webpages most commonly presented to the US public for abortion pill searches on Google and to evaluate the quality of those webpages providing textual educational content, with the goal of contributing to a better understanding of online abortion pill information. Using the FPWQAT designed for this research, we found that only the top webpage for “abortion pill” searches was of high-quality when accounting for both quality of information and user experience. The subsequent top webpage results were of notably lower quality, presenting limited accurate clinical information in less useable formats. Three of these pages presented abortion pill disinformation, a finding corroborated by past research showing that anti-abortion webpages (specifically CPC webpages) include a wealth of disinformation on sexual and reproductive health concerns [12–14, 49, 50]. Our findings call attention to the challenges for users who sought reliable abortion information online, as the webpage quality of common search results may have hindered rather than facilitated informed reproductive choice and access to the abortion pill.

The high score of the plannedparenthood.com abortion pill webpage, and its ranking as both the first search result and again as the third result out of the top 10 pages (not assessed separately as it was captured in the page 1 assessment), leads us to conclude that around 45% of clicks from information seekers likely lead to complete and balanced information on the abortion pill through Google [38]. Nonetheless, anti-abortion webpages, which all scored poorly on our assessment, constituted several of the top pages assessed. Even accounting for decreasing click-throughs going down the ranking of results, these anti-abortion webpages could have accounted for around 30% of clicks for “abortion pill” searches—meaning that around 30% of searchers would click on (and likely consume information from) those webpages. The wikipedia.com page, which received about 3% of clicks, had a moderately useable format and provided some accurate factual information, but had very little accurate clinical information. This is surprising, as Wikipedia is the largest and most popular general reference resource on the internet and has been found to not significantly differ from expert generated webpages in accuracy and medical completeness of medical in past studies [51, 52]. While the Wikipedia.com page was not overtly anti-abortion, the quality of that page also poses a challenge to access reliable abortion information and services for users. We hence conclude that the majority of top webpages in August of 2018 that provided textual abortion pill information were not high-quality, with around one-third of clicks likely leading to incomplete and not highly useable information on the abortion pill.

The API used for our analysis did not allow us to examine differences in webpage search results within the US. However, past research has demonstrated that search results and access to information are not equivalent for people in different areas of the US [11]. Prior findings highlight that while search results hindering abortion access are a concern across the US, people who face more barriers to abortion access likely also face additional barriers to finding reliable information online [3]. This is of greater concern when considering that users who face barriers to accessing in-person healthcare services are more likely to turn to the internet for health information than people who do not face the same barriers [53]. As we consider the implications of inequitable access to information and variable reliance upon it, we must consider that the onus for determining the credibility of online information generally falls on the individual user, who is operating in a context where the available information is vast and constantly changing [54]. This leaves the individual with the difficult task of discerning credible information from the profusion of results, a task that some users, specifically those with more education, may have the skills to more effectively carry out than others [55]. For abortion pill searchers, the confluence of barriers may be exacerbated by limited or unreliable internet access, as well as the particular stigmas and disinformation challenging individuals seeking abortion pill information.

The presence of anti-abortion webpages within top search results is situated within a longer history of anti-abortion webpages on Google’s search interface. Google has often faced pressure from reproductive rights advocates to more responsibly handle the presentation of misleading webpages to users seeking reliable reproductive health information and services on their search interface. Over the past seven years, Google has faced intensive scrutiny and investigation regarding their policies around advertisements and presentation of results for abortion searches. Though Google has taken steps to improve the regulation and labeling of results on their search engine, little real progress has been made to protect users from deceptive abortion search results [56–59]. There is opportunity and precedent for restructuring search outputs by changing algorithms, effectively improving the quality of results to benefit the public. For instance, for the search term “lesbian” (*lesbienne)* in France, Google has changed their algorithm to include more informational content and less pornography in top results, spurred by a popular news investigation and social pressure [60]. If these positive changes were integrated into Google’s algorithm for “abortion pill,” far more searchers in the US could access higher quality results. While the decisions surrounding Google’s algorithm are a space of continued controversy and conflict, our findings underline the need for improvement in search outputs. There is also opportunity to encourage users and healthcare providers to use trusted and validated webpages as sources of abortion pill information

Our analysis was structured to be comprehensive and rigorous, providing meaningful information for an area of online health information that is both highly contentious and vital to informed reproductive decision-making in our current US context, but there were limitations to our approach. Using Google’s Custom Search API to get top webpages is a novel approach that provides insights into online abortion pill information, but this assessment only accounted for the top text-based educational webpages for “abortion pill” searches on the date of our query (06 August 2018). While our choice to restrict our assessment to these pages provided focused results and facilitated assessment using established health information quality criteria, we only assessed five of the top 10 pages. To address the resulting gaps in our understanding of abortion pill webpage results, further research could focus on other types of top pages, taking different assessment approaches to look at videos or news articles. Additionally, as discussed above our methodology only allowed us to explore top webpages for the entire US, making it impossible to account for any differences in top webpages and quality of results for smaller geospatial areas.

Quality assessment of top webpages at additional time points and for other search queries in sexual and reproductive health—including other queries for the abortion pill, surgical abortion, and methods of contraception—is also needed. The FPWQAT developed for this study was designed to facilitate this type of broader assessment by research teams and can be applied to webpages presenting information on a range of methods of contraception and abortion, with comparability across content areas that allows for connections across methods despite differences in clinical and factual information. Also notable, during this assessment it was clear based on webpage reviews that online resources strike a balance between providing comprehensive information and content easily consumed by users. For our assessment, comprehensive, accurate information on a webpage was high quality, as we saw online resources as a potential stand-in for in-person counselling with a provider, particularly in hostile states that drive people to seek online information out of fear or shame of seeking services and pose multiple access barriers [9–12]. As a result, some pages may have provided accurate basic information on the abortion pill and scored poorly. Our focus on comprehensiveness also resulted in a lengthy assessment intended as a research tool, as a result to FPWQAT has limited utility for the public without revision. Additionally, in this analysis the FPWQAT was applied by a team with strong internal consensus. This provided results with high levels of agreement, but these results may also reflect the biases of a pro-choice team immersed in sexual and reproductive health research.

Furthermore, as our aim was to create a tool to assess overall webpage quality, we did not capture any information about the sentiment of content in this analysis. For abortion particularly, much of the language used by advocacy groups on both sides can be highly charged and strategically designed to illicit emotional response. Understanding the sentiment of the language used online about abortion and the emotional responses it evokes in consumers is an important area for future exploration. Furthermore, we did not directly assess user experience in this analysis by speaking to users, rather we used validated criteria for overall user experience of webpages and health information. We need to explore user’s perceptions of these pages and how they interact with other aspects of experience, from sexual education to social networks and beyond. Past research has found that the perspectives of patients differ from those of researchers in assessing information online and emphasized the need for integrating the views and preferences of patients in the interpretation and assessment of online information [61, 62], as has been done with pregnancy information already [63].

## Conclusions

This analysis identified the top webpages for abortion pill searches on Google as of August 2018 and provided meaningful insights into the quality of those webpages using a novel assessment tool. Our findings show plannedparenthood.com was the only top webpage providing accurate information in a useable format, and we can infer that almost half of estimated click-throughs led to this reliable source of abortion pill information. All other top webpage results provided lower quality information in less useable formats, and three of five presented disinformation aligned with the anti-abortion interests of the website owners. The reality of anti-abortion pages constituting the majority of top informational abortion pill search results points to challenges for users in finding high-quality information on abortion in the current context of socio-political polarization and stigma around abortion. As people face additional impediments to accessing in-person health services and the internet serves as an increasingly important pathway to health information and services, supporting consumer access to credible online abortion pill information is central to informed reproductive choice. Advocates and policymakers should continue to push for resources to promote consumer discernment of credible online information and the prioritization of presenting Google searchers across the US with consistently credible and reliable content, particularly when critical health decisions and reproductive choices are at stake.

## Supporting information

S1 Appendix Family planning webpage quality assessment tool. (PDF)

S1 Table Abortion pill top webpages full FPWQAT scoring results. (PDF)
