# Supplementary material for: Quality of top webpages providing abortion pill information for Google searches in the USA: An evidence-based webpage quality assessment
Source: PLoS One. 2021 Jan 21;16(1):e0240664. doi: 10.1371/journal.pone.0240664 (PMC7819599; doi:10.1371/journal.pone.0240664)
Supplement: S1 Appendix — (PDF) [file pone.0240664.s001.pdf]

S1 Appendix:

Family Planning Webpage Quality Assessment Tool

Wallace Center for MCAH Research

Tool to Assess the Quality of Webpages Providing Text-Based Sexual and Reproductive Health Education

Created by E. Pleasants, K. Weidert, N. Prata, and S. Guendelman as a part of *Understanding Consumer Behavior to Advance Reproductive Health in the United States,* funded by the David and Lucille Packard Foundation and the Wallace Center for MCAH Research

Purpose: To assess the quality of webpages, as determined by their overall credibility and usability, and the accuracy of sexual and reproductive health information presented, as determined by the alignment of information presented with best-practice information on specific contraceptive methods, options counselling provision, and abortion. This assessment will determine what top webpages are doing well and any notable shortcomings or gaps in information.

Audience: Researchers and practitioners with an interest in online sexual and reproductive health information

Date of assessment (mm/dd/yyyy): ___________________________________

Primary search term:

( ) Birth control
( ) Abortion

Birth control follow-up search term

( ) Birth control patch
( ) IUD birth control
( ) Birth control pills
( ) Birth control implant
( ) Birth control shot

Abortion follow-up search term

( ) Partial birth abortion
( ) Abortion clinics
( ) Abortion pill
( ) Abortion clinic
( ) Abortion facts

Webpage (full url): ____

Section I should be used for sites that discuss specific methods of contraception or abortion.

Section II should be used for all sites to assess overall quality.

Before beginning the assessment, review the entire webpage at least once to get familiar with layout, formatting, and content. Next, begin to assess the webpage based on the criteria in Section I and then Section II. All information is assessed based on if it is present and accurate based on the assessment questions.

Directory of sections for Family Planning Webpage Quality Assessment Tool

Section I: Accuracy of Information by Topic……………………...…3

Combined Oral Contraceptive Pill……………………………..……3
Contraceptive Pill: Clinical Information ……….….………3
Combined Oral Contraceptive Pill: Facts………………..……7

Intra Uterine Device (IUD) ……………………………….………8
IUD: Clinical Information……………………………….………8
IUD: Facts……………………………………………………12

Contraceptive shot/injectable/depo (Provera) ……………….…....13
Contraceptive Injection: Clinical Information ………………13
Contraceptive Injection: Facts…………………………..…17

Contraceptive Patch…………………………………..………18
Contraceptive Patch: Clinical Information………..………18
Contraceptive patch: Facts…………………………..……22

Contraceptive Implant…………………………………..………23
Contraceptive Implant: Clinical Information ………….……23
Contraceptive Implant: Facts…………………………..……26

Medication abortion (abortion pill) ……………………….………28
Medication abortion: clinical information………..……28
Medication abortion: facts…………………………………30

Surgical Abortion…………………………………………………31
Surgical Abortion: clinical information …..………………31
Surgical abortion: facts…………………………………..…33

Section II: User Experience of Webpage……………………..……35

Section I: Accuracy of Information by Topic

Combined Oral Contraceptive Pill

This section assesses whether the page accurately presents each piece of information regarding combined oral contraceptive (COC) pills

<u>Contraceptive Pill: Clinical Information</u>

1) Webpage correctly identifies all of the following contraindications:
    - Pregnancy or suspicion of pregnancy
    - Over 35 and smoker
    - History of hypertension
    - History of DVT
    - Known thrombogenic mutations
    - Ischemic heart disease (current or history of) or stroke (history of)
    - Complicated valvular heart disease
    - Migraines with aura
    - Current breast cancer
    - Diabetes
    - Less than 6 weeks postpartum
    - Benign or malignant liver tumors, active liver disease

    Potential OC users should be carefully screened to minimize possible risks. Age, health history, and smoking are extremely important

    Correct identification of contraindications:

    ( ) Yes *(skip to 2)*
    ( ) No

    1a) Contraindications incorrectly described:

    ____
    ____
    ____
    ____

    1b) Contraindications not identified:

    ____
    ____
    ____
    ____

2) **Webpage correctly describes required daily use of the pill.**

    Must be used daily at the same time

    ( ) Yes
    ( ) No

3) **Webpage correctly describes that the pill is not immediately effective and must be used consistently for effective pregnancy prevention, as well as what to do if one or more pills is missed.**

Takes ~7 days to effectively prevent pregnancy alone, use alternative contraceptive method in the meantime.

If you miss taking one pill:
- Take the late or missed pill as soon as possible.
- Continue taking the remaining pills at the usual time (even if it means taking two pills on the same day).
- No additional contraceptive protection is needed.
- Emergency contraception is not usually needed but can be considered if hormonal pills were missed earlier in the cycle or in the last week of the previous cycle

If you miss two or more pills:
- Take the most recent missed pill as soon as possible (any other missed pills should be discarded).
- Continue taking the remaining pills at the usual time (even if it means taking two pills on the same day).
- Use back-up contraception (e.g., condoms) or avoid sexual intercourse until hormonal pills have been taken for 7 consecutive days.
- If pills were missed in the last week of hormonal pills (e.g., days 15-21 for 28-day pill packs):
  - Omit the hormone-free interval by finishing the hormonal pills in the current pack and starting a new pack the next day.
  - If unable to start a new pack immediately, use backup contraception (e.g., condoms) or avoid sexual intercourse until hormonal pills from a new pack have been taken for 7 consecutive days
- Emergency contraception should be considered if hormonal pills were missed during the first week and unprotected sexual intercourse occurred in the previous 5 days.
- Emergency contraception may also be considered at other times as appropriate.

Correctly describes required daily use:

( ) Yes
( ) No

4) **Webpage correctly described how the COCP is used.**

Oral pill to be taken daily at the same time to prevent pregnancy. Some people take them 21 days on, 7 days off; others take pills in continuous dose for 3 months to reduce menstrual periods to 4 times a year

( ) Yes
( ) No

5) **Webpage makes it clear that the pill is reversible and not taking it can result in pregnancy.**

If you stop taking the pill or even miss more than one pill you are at risk/able to get pregnant

( ) Yes
( ) No

6) **Webpage clearly states how effective the pill is when used by most women, reflecting that while it is effective at preventing pregnancy it is not a highly effective method with typical use.**

With typical use—meaning that the method may not always be used consistently or correctly—9 women out of 100 (9%) will become pregnant during the first year of using these methods. With perfect use—meaning that the method is used consistently and correctly each time—fewer than 1 woman out of 100 will become pregnant during the first year

( ) Yes
( ) No

7) **Webpage states that the combined oral contraceptive pill is a hormonal method of contraception and contains estrogen.**

Cannot be used while breastfeeding or by women with high stroke risk

( ) Yes
( ) No

8) **Webpage states that the pill is a safe method of contraception.**

( ) Yes
( ) No

9) **Webpage accurately describes risks associated with use of COCPs.**
- It is uncommon, but some women develop high blood pressure when taking the pill.
- It is uncommon, but the use of the combined pill increases the risk of blood clots, heart attack, and stroke in some women
- Consistent evidence of increased risk of breast and cervical cancer

Risks accurately described:

( ) Yes *(skip to 10)*
( ) No

9a) Risks incorrectly described:

____
____
____
____

9b) Risks not described:

____
____
____
____

10) **Webpage accurately describes possible side effects of COCP use.**

- Can cause nausea
- Can cause changes in mood
- Can cause breast tenderness

- Can cause headaches

Possible side effects accurately described:

( ) Yes *(skip to 11)*
( ) No

10a) Side effects incorrectly described:

____
____
____
____

10b) Side effects not described:

____
____
____
____

11) **Webpage correctly describes the non-contraceptive benefits of COCPs.**

- May improve acne
- May decrease the risk of cancer of the uterus, ovary, and colon.
- Can be used to treat certain disorders that cause heavy bleeding and menstrual pain, such as *fibroids* and *endometriosis.* Used continuously, they can reduce the frequency of migraines associated with menstruation (although they should not be used if you have migraines with *aura*).

Non-contraceptive benefits accurately described:

( ) Yes *(skip to 12)*
( ) No

11a) Non-contraceptive benefits incorrectly described:

____
____
____
____

11b) Non-contraceptive benefits not described:

____
____
____
____

12) **Webpage clearly states that the pill does not protect against STIs.**

Alternative method, such as condoms, should be used for STI protection

( ) Yes
( ) No

13) **Webpage clearly describes the possible effects of COCPs on menstrual bleeding.**

- May make your period more regular, lighter, and shorter.
- Can help reduce menstrual cramps.

( ) Yes
( ) No

14) **Webpage states that certain medications make the pill less effective.**

Can make reference to speaking to a provider about the specific meds or list them: Rifampin, some anti-seizure medications, and supplements (such as St. John's Wort).

( ) Yes
( ) No

<u>Combined Oral Contraceptive Pill: Facts</u>

15) **Webpage makes it clear that getting the pill requires a health care provider visit and prescription in most states.**

( ) Yes
( ) No

16) **Webpage correctly describes how much the pill usually costs, clearly stating on the page being assessed (not elsewhere on the page) that many people can get the pill at low cost or free depending on their insurance and where they live.**

Can provide further details on cost and accurately representing the following information:

In 2010, the Affordable Care Act (ACA) took state laws further by requiring most private plans (including self-funded, small and large group, and individual plans) to cover a wide range of recommended preventive services, without cost to policyholders. In 2011, HRSA, following recommendations issued by the Institute of Medicine, added that all FDA-approved contraceptive methods and patient counseling for women with reproductive capacity, as prescribed by a health care provider be included as a preventive service. The policy requires that most private health insurance plans cover at least one form of each of the 18 FDA-approved contraceptive methods for women as prescribed without cost sharing. This means that plans must cover at least one of each of the three different types of oral contraceptives – the combined pill, the progestin-only pill and the continuous use pill – though it is up to an insurer's discretion using reasonable medical management practices whether to cover a brand name or generic contraceptive if both are available.

Cost of the pill accurately described:

( ) Yes
( ) No

17) **This webpage does not make any incorrect claims about the pill.**

If it makes any of the following claims, select NO below:

- Causes weight gain
- Causes acne
- Increases breast cancer risk
- Can cause future infertility

- Is dangerous for your health
- Causes birth defects
- Causes promiscuity
- Lowered sex drive

Makes false claims about the pill:

( ) Yes
( ) No *(skip to 18)*

17a) False/erroneous claims made:

____
____
____
____

Intra Uterine Device (IUD)

This section assess whether the page accurately presents each piece of information regarding both copper and progestin-only IUDs.

<u>**IUD: Clinical Information**</u>

18) **Webpage correctly identifies all of the following contraindications to IUD use:**
   - Known or suspected pregnancy
   - Current cervical infection
   - Recent PID
   - Cancer of the genital tract
   - Uterine anomalies
   - Abnormal uterine bleeding
   - History of breast cancer – hormonal only
   - Wilson's disease or copper allergy – copper only
   - Recent postpartum or post abortion infection

Correct identification of contraindications:

( ) Yes (*skip to 19)*
( ) No

18a) Contraindications incorrectly described:

____
____
____
____

18b) Contraindications not described:

____
____
____
____

19) **Webpage correctly describes that IUDs are long acting and their period of efficacy.**

3-5 years depending on brand for hormonal, and copper IUD approved for up to 12

( ) Yes
( ) No

20) **Webpage correctly describes the period of time until each type of IUD discussed begins to effectively prevent pregnancy.**

The copper IUD also is the most effective form of emergency contraception. It is immediately effective at preventing pregnancy.

Hormonal IUD takes ~7 days to effectively prevent pregnancy, use alternative protection in the meantime

( ) Yes
( ) No

21) **Webpage correctly describes how IUDs are used.**

The copper IUD is a small device that is shaped in the form of a "T." Your doctor places it inside the uterus to prevent pregnancy.
The LNG IUD is a small T-shaped device. It is placed inside the uterus by a doctor. It releases a small amount of progestin each day to keep you from getting pregnant.

( ) Yes
( ) No

22) **Webpage makes it clear that IUDs are reversible methods of contraception.**

If you wish to get pregnant or if you want to stop using it, you can have the IUD removed. Using an IUD does not affect your ability to get pregnant in the future

( ) Yes
( ) No

23) **Webpage clearly states that IUDs are a method of contraception "most effective" at preventing pregnancy (exact language does not have to be used, but should include percent effective).**

Less than 1 in 100 women get pregnant in first year of use (>99% effective)

( ) Yes
( ) No

24) **Webpage states if the IUD is hormonal and what hormones it contains for each type discussed:**
The copper IUD does not contain hormones, and that the hormonal IUD is progestin only. The Copper T IUD does not contain hormones or interact with medicines. Both can be inserted immediately postpartum, post abortion or post-miscarriage and used while breastfeeding.

( ) Yes
( ) No

25) **Webpage states that IUDs are a safe method of contraception.**

( ) Yes
( ) No

26) **Webpage accurately describes the risks associated with each type of IUD use discussed.**

- In a small number of women, the IUD may come out of the uterus. The risk is higher in teenagers, women with heavy menstrual bleeding, and women who have an IUD inserted immediately after childbirth. If the IUD comes out, it is no longer effective. You may be able to have a new IUD placed.
- The IUD can go through the wall of the uterus during placement. This usually does not cause any major health problems, but the IUD will need to be removed. It is rare and occurs in only about 1 out of every 1,000 placements.
- Pelvic inflammatory disease (PID) after IUD insertion happens very rarely. Using an IUD does not by itself increase the risk of PID. Women with an undiagnosed STI at the time of IUD insertion are more likely to develop PID than women without an STI. If you are at risk of STIs, you may be screened before you get an IUD.
- Rarely, pregnancy may occur while a woman is using an IUD. If pregnancy occurs, and you wish to continue the pregnancy, the IUD should be removed if your ob-gyn or other health care professional can see the IUD in the cervix or if the strings are visible. If the IUD remains in place during pregnancy, there are increased risks of miscarriage and infection.
- In the rare case that a pregnancy occurs with the IUD in place, there is a higher chance that it will be an ectopic pregnancy. This is a serious condition that needs medical attention right away.

Risks accurately described:

( ) Yes *(skip to 27)*
( ) No

26a) Risks incorrectly described:

____
____
____
____

26b) Risks not described:

____
____
____
____

27) **Webpage accurately describes possible side effects of use of each types of IUD discussed.**

- Placement of the IUD may cause some temporary discomfort. Taking over-the-counter pain relief medication such as ibuprofen before or after placement may help.
- The IUD has strings made of thin plastic threads. Your ob-gyn or other health care professional will use the strings to remove the IUD when you decide to stop using it. The strings should not bother you, but in rare cases your sexual partner may feel them during sex. If this occurs and it is a concern, your ob-gyn or other health care professional may be able to trim the strings
- Some women also may experience other side effects, including headaches, nausea, breast tenderness, and mood changes with hormonal IUDs

Possible side effects accurately described:

( ) Yes *(skip to 28)*
( ) No

27a) Side effects incorrectly described:

____
____
____
____

27b) Side effects not described:

____
____
____
____

28) **Webpage correctly describes non-contraceptive benefits of IUDs.**

For levonorgestrel-releasing intrauterine system:

- Controls heavy menstrual bleeding and anemia
- Reduces endometrial hyperplasia and cancer
- Reduces rates of endometrial polyps in users of tamoxifen and alleviates pain associated with endometriosis and adenomyosis

For Depot medroxyprogesterone acetate:

- Controls crises of pain associated with sickle cell disease and endometriosis.

Copper intrauterine device users have:

- Reduced rates of endometrial and cervical cancer.

Non-contraceptive benefits accurately described:

( ) Yes *(skip to 29)*
( ) No

28a) Non-contraceptive benefits incorrectly described:

____
____
____
____

28b) Non-contraceptive benefits not described:

____
____
____
____

29) **Webpage clearly states that IUDs do not protect against STIs.**

Alternative method, such as condoms, should be used for STI protection

( ) Yes
( ) No

30) **Webpage clearly states the effects of each type of IUD discussed on menstrual bleeding.**

- When a woman uses an IUD, changes in menstrual bleeding are normal and not harmful. With the copper IUD, painful periods and bleeding may increase during the first months of use. Over-the-counter pain relievers may be used for pain and bleeding
- Hormonal IUDs may cause frequent spotting, more days of bleeding, and heavier bleeding in the first months of use. Over time, the amount of menstrual bleeding and the length of your menstrual period usually decrease. Menstrual pain also usually decreases. For some women using a hormonal IUD, menstrual bleeding stops completely

( ) Yes
( ) No

<u>IUD: Facts</u>

31) **Webpage makes it clear that getting an IUD requires an appointment with healthcare provider for exam and insertion to start the method.**

An obstetrician–gynecologist (ob-gyn) or other health care professional must place an IUD. He or she will review your medical history and will perform a pelvic exam. The IUD will be guided through the vagina and the cervix and then into the uterus

( ) Yes
( ) No

32) **Webpage correctly describes how much an IUD usually costs, clearly stating on the page being assessed (not elsewhere on the page) that many people can get IUDs at low cost or free depending on their insurance coverage and where they live.**

The costs of IUDs have been a barrier to its use, for both patients and providers. Prices for an IUD typically range between $500 and $1,000, in addition to provider visits for insertion, removal and confirmation that the device was properly placed. The ACA includes a requirement that most private insurance plans must cover at least one type of all 18 FDA-approved contraceptive methods for women as prescribed without cost sharing. This means that most private plans (small and large group, self-funded, and individually purchased plans) must cover the copper IUD and at least one hormonal IUD at no cost to policy holders.

( ) Yes
( ) No

33) **This webpage does not make any incorrect claims about the IUD.**

If it makes any of the following claims, select NO below:

- Increased risk of PID or STI
- Increased risk of ectopic pregnancy
- Abortifacient
- Decreases fertility or causes infertility
- Recommended for women with at least one child or after family is complete (including claim they are not appropriate for teens)
- Recommended for women with low risk of STIs or in a monogamous relationship
- Dalkon Shield history is relevant for today's IUDs

Makes false claims about IUDs:

( ) Yes
( ) No *(skip to 34)*

33a) False/erroneous claims made:

____
____
____
____

Contraceptive shot/injectable/depo (Provera)

This section assesses whether the page accurately presents each piece of information regarding contraceptive injections

<u>**Contraceptive Injection: Clinical Information**</u>

34) **Webpage correctly identifies all of the following contraindications:**

- Known or suspected pregnancy
- Current breast cancer
- CVD risk factors (older age, smoking hypertension)
- Vascular disease
- Current or history of ischemic heart disease or stroke
- SLE
- Rheumatoid arthritis
- Migraine with aura
- Unexplained vaginal bleeding
- Diabetes (only if nephropathy, retinopathy, neuropathy, or other vascular disease is present, or the duration of diabetes is >20 years)
- Severe cirrhosis
- Malignant liver tumor
- Certain antiretroviral and anticonvulsant medications (some are designated category 2)

Correct identification of contraindications:

( ) Yes *(skip to 35)*
( ) No

34a) Contraindications incorrectly described:

____
____
____
____

34b) Contraindications not described:

____
____
____
____

35) **Webpage correctly describes required injection approximately every three months.**

Progestin only 3-month (12 week) injectable that is administered by a medical provider. Can be administered up to two weeks early or two weeks late

( ) Yes
( ) No

36) **Webpage correctly describes that the injection is not immediately effective and must be used consistently for effective pregnancy prevention.**

Takes ~7 days to effectively prevent pregnancy alone unless administered within the first 7 days of a menstrual period. If administered at any other time use an alternative contraceptive method in the meantime.

( ) Yes
( ) No

37) **Webpage correctly describes how the contraceptive injection is used.**

Women get shots of the hormone progestin in the buttocks or arm every three months from their doctor.

( ) Yes
( ) No

38) **Webpage makes it clear that the injection is reversible but that it may take time after discontinuing use to get pregnant, particularly with prolonged use.**

( ) Yes
( ) No

39) **Webpage clearly states how effective the injection is when used by most women, reflecting that while it is effective at preventing pregnancy it is not a highly effective method with typical use, particularly when shots are not received on schedule.**

With typical use—meaning that the method may not always be used consistently or correctly—of 100 women who use this method each year, about six may become pregnant, 94% effective. Pregnancy risk is lower among women who get their shot on schedule

( ) Yes
( ) No

40) **Webpage makes it clear that the injection is a hormonal method of contraception but only contains progestin.**

Can be used by smokers over 35, women who are breastfeeding and others who cannot use estrogen-containing method

( ) Yes
( ) No

41) **Webpage states that the injection is a safe method of contraception.**

( ) Yes
( ) No

42) **Webpage accurately describes risks associated with use of the contraceptive injection.**

- Some evidence that use can result in loss of bone mass over time, though it is recommended that providers do not allow concerns over this to limit their prescription
- Delayed return to fertility (10 months after last injection median time)
- Inconclusive whether women using depo are at increased risk of HIV

Risks accurately described:

( ) Yes *(skip to 43)*
( ) No

42a) Risks incorrectly described:

____
____
____
____

42b) Risks not described:

____
____
____
____

43) **Webpage accurately describes possible side effects of the contraceptive injection.**

- Can cause breast tenderness
- Can cause weight gain
- Can cause nausea
- Can cause headaches
- Can cause injection page reactions
- Can cause mood changes or depression

Possible side effects accurately described:

( ) Yes *(skip to 44)*
( ) No

43a) Side effects incorrectly described:

____
____
____
____

43b) Side effects not described:

____
____
____
____

44) **Webpage correctly describes the non-contraceptive benefits of the injection.**

- Reduced risk of endometrial cancer by up to 80% with benefits continuing after discontinuation
- Reduced risk of PID
- Reduced risk of leiomyomata
- Reduced severity and number of crises in people with sickle cell anemia
- Safe/beneficial for people with epilepsy

Non-contraceptive benefits accurately described:

( ) Yes *(skip to 45)*
( ) No

44a) Non-contraceptive benefits incorrectly described:

____
____
____
____

44b) Non-contraceptive benefits not described:

____
____
____
____

45) **Webpage clearly states that the injection does not protect against STIs.**

Alternative method, such as condoms, should be used for STI protection

( ) Yes
( ) No

Contraceptive Injection: Facts

46) **Webpage makes it clear that getting the injection requires a provider visit and prescription in most states, and a visit every 12 weeks.**

( ) Yes
( ) No

47) **Webpage correctly describes how much the injection usually costs, clearly stating on the page being assessed (not elsewhere on the page) that many people can get it at low cost or free depending on their insurance and where they live.**

One injection lasts for three months and costs $35 to $100 (excluding office exam fees). Generic should be, and often is, covered without cost sharing and is available in most pharmacies. Can get the shot prescribed and administered by pharmacists in some states

( ) Yes
( ) No

48) **Webpage clearly describes the possible effects of the injection on menstrual bleeding.**

- Initially can cause spotting or bleeding between periods, rarely heavy bleeding
- Shorter, lighter periods or no periods at all
- Reduced menstrual cramping

( ) Yes
( ) No

49) **This webpage does not make any incorrect claims about the injection.**

If it makes any of the following claims, select NO below:

- It causes abnormal babies later
- Causes abortion
- Causes cancer
- Causes infertility
- Cannot be used while breastfeeding, harms breastmilk
- Effects sexual desire

Makes false claims about the injection:

( ) Yes
( ) No *(skip to 50)*

49a) False/erroneous claims made:

____
____
____
____

Contraceptive Patch

This section assesses whether the page accurately presents each piece of information regarding the contraceptive patch

<u>Contraceptive Patch: Clinical Information</u>

50) **Webpage correctly identifies all of the following contraindications for patch use:**
- Pregnancy or suspicion of pregnancy
- Over 35 and smoker
- History of hypertension
- History of DVT
- Known thrombogenic mutations
- Ischemic heart disease (current or history of) or stroke (history of)
- Complicated valvular heart disease
- Migranes with aura
- Current breast cancer
- Diabetes
- Less than 6 weeks postpartum
- Benign or malignant liver tumors, active liver disease

Potential patch users should be carefully screened to minimize possible risks. Age, health history, and smoking are extremely important

Correct identification of contraindications:

( ) Yes *(skip to 51)*
( ) No

50a) Contraindications incorrectly identified:

____
____
____
____

50b) Contraindications not identified:

____
____
____
____

51) **Webpage correctly describes monthly use of the patch, with three weeks of wearing a patch and one week without for menstrual period recommended if you want a period or continuously if you do not want a period.**

( ) Yes
( ) No

52) **Webpage correctly describes that the patch is not immediately effective on initial use and must be timed correctly for protection during later use, including what to do if the patch comes off.**

If you start using the patch on the first day of your period, and up to and including the fifth day of your period, you'll be protected from pregnancy straight away. If you start using it on any other day, you need to use an additional form of contraception, such as condoms for the first 7 days.

If the patch does fall off, what you need to do depends on how long it has been off, and how many days the patch was on before it came off.
If it's been off for less than 48 hours:

- stick it back on as soon as possible if it's still sticky
- if it's not sticky, put a new patch on (don't try to hold the old patch in place with a plaster or bandage)
- continue to use your patch as normal and change your patch on your normal change day
- you're protected against pregnancy and won't need additional contraception if it was on correctly for 7 days before it came off
- but if the patch fell off after using it for 6 days or less, use additional contraception, such as condoms, for 7 days

If it's been off for 48 hours or more, or you're not sure how long:

- apply a new patch as soon as possible and start a new patch cycle (this will now be day one of your new cycle)
- use additional contraception, such as condoms, for the next 7 days
- see a GP or nurse for advice if you've had unprotected sex in the previous few days as you may need emergency contraception

( ) Yes
( ) No

53) **Webpage correctly describes how the contraceptive patch is used.**

This skin patch is worn on the lower abdomen, buttocks, or upper body (but not on the breasts). This method is prescribed by a doctor. It releases hormones progestin and estrogen into the bloodstream and each patch is designed to be worn for three weeks, but has an additional two days it can be used and serum hormone levels maintained.

( ) Yes
( ) No

54) **Webpage makes it clear that the patch is reversible and not wearing it according to schedule can result in pregnancy.**

( ) Yes
( ) No

55) **Webpage clearly states how effective the patch is when used by most women, reflecting that while it is effective at preventing pregnancy it is not a highly effective method with typical use.**

With typical use—meaning that the method may not always be used consistently or correctly—about nine get pregnant (91% effective).

( ) Yes
( ) No

56) **Webpage states that the patch is a hormonal method of contraception containing estrogen, and at higher levels than other methods.**

Cannot be used while breastfeeding or by women with high stroke risk.

( ) Yes
( ) No

57) **Webpage states that the patch is a safe method of contraception.**

( ) Yes
( ) No

58) **Webpage accurately describes the risks associated with use of the patch.**

- It is uncommon, but some women develop high blood pressure when using the patch
- It is uncommon, but the use of the patch increases the risk of blood clots, heart attack, and stroke in some women
- Consistent evidence of increased risk of breast and cervical cancer
- Slight increase in risk of VTE compared to COCs

Risks accurately described:

( ) Yes *(skip to 59)*
( ) No

58a) Risks incorrectly described:

____
____
____
____

58b) Risks not described:

____
____
____
____

59) **Webpage accurately describes the possible side effects of using the patch.**

- Can cause skin irritation
- Can cause breast tenderness
- Can cause headaches
- Can cause nausea/vomiting
- Can cause mood changes

Possible side effects accurately described:

( ) Yes *(skip to 60)*
( ) No

59a) Side effects incorrectly described:

____
____
____
____

59b) Side effects not described:

____
____
____
____

60) **Webpage correctly describes the non-contraceptive benefits of using the patch.**

- May improve acne.
- May decrease the risk of cancer of the uterus, ovary, and colon.
- Can be used to treat certain disorders that cause heavy bleeding and menstrual pain, such as fibroids and endometriosis.
- Can also be used to prevent development of ovarian cysts
- Used continuously, they can reduce the frequency of migraines associated with menstruation (although they should not be used if you have migraines with aura).

Non-contraceptive benefits accurately described:

( ) Yes *(skip to 61)*
( ) No

60a) Non-contraceptive benefits incorrectly described:

____
____
____
____

60b) Non-contraceptive benefits not described:

____
____
____
____

61) **Webpage clearly states that the patch does not protect against STIs.**

Alternative method, such as condoms, should be used for STI protection

( ) Yes
( ) No

62) **Webpage clearly describes the possible effects of the patch on menstrual bleeding.**

- Can cause irregular/breakthrough bleeding
- Can make your period more regular, lighter, and shorter
- Can help reduce menstrual cramps

( ) Yes
( ) No

63) **Webpage states that certain medications make the patch less effective.**

Can make reference to speaking to a provider about the specific meds or list them: Rifampin, some anti-seizure medications, and supplements (such as St. John's Wort).

( ) Yes
( ) No

64) **Webpage clearly states that in some cases the patch has been found less effective for women of higher body weights (198 pounds, does not have to state this exact weight but should mention potential reduced efficacy).**

( ) Yes
( ) No

<u>Contraceptive patch: Facts</u>

65) **Webpage makes it clear that getting the patch requires a provider visit and prescription in most states.**

( ) Yes
( ) No

66) **Webpage correctly described how much the patch usually costs, clearly stating on the page being assessed (not elsewhere on the page) that many people can get the patch at low cost or free depending on their insurance and where they live.**

Three patches (a 30-day supply) costs about $15 to $80, generally available at pharmacies and generic covered with no cost sharing

( ) Yes
( ) No

67) **This webpage does not make any incorrect claims about the patch.**

If it makes any of the following claims, select NO below:

- Causes weight gain
- Causes acne
- Can cause future infertility
- Is dangerous for your health
- Causes birth defects
- Causes promiscuity

Makes false claims about the patch:

( ) Yes
( ) No *(skip to 68)*

67a) False/erroneous claims made:

____
____
____
____

Contraceptive Implant

This section assesses whether the page accurately presents each piece of information regarding the contraceptive implant

<u>Contraceptive Implant: Clinical Information</u>

68) **Webpage correctly identifies all of the following contraindications:**

- Current breast cancer
- Current or history of ischemic heart disease or stroke
- SLE
- Migraine with aura
- Unexplained vaginal bleeding
- Breast cancer in the past
- Severe cirrhosis
- Malignant liver tumor

Correct identification of contraindications:

( ) Yes *(skip to 69)*
( ) No

68a) Contraindications incorrectly identified:

____
____
____
____

68b) Contraindications not identified:

____
____
____
____

69) **Webpage states that the contraceptive implant is long acting, effectively preventing pregnancy for up to three years.**

( ) Yes
( ) No

70) **Webpage correctly describes that the implant is not immediately effective unless inserted during the first 5 days of a menstrual period. If inserted at any other time, another method of contraception such as condoms should be used for the first 7 days of implant use before pregnancy protection begins.**

( ) Yes
( ) No

71) **Webpage correctly describes how the contraceptive implant is used.**

The implant is a single, thin rod that is inserted under the skin of a women's upper arm. Insertion is done by a health care provider in their office; they numb the skin in the area where the implant is inserted and insert it using an applicator. The rod contains a progestin that is released into the body over 3 years. Removal involves a minor surgical procedure where a healthcare provider numbs the skin around the implant, makes and incision, and removes the implant.

( ) Yes
( ) No

72) **Webpage makes it clear that the implant is reversible and removing the implant results in rapid return to normal fertility and risk of pregnancy.**

After implant is removed, most women (94%) ovulate by 3 months, the majority within 3 weeks

( ) Yes
( ) No

73) **Webpage makes it clear that the implant is a very effective method of contraception falling in the "most effective" category (exact language does not have to be use but percent effective should be presented).**

Less than 1 in 100 women get pregnant in first year of use (>99% effective)

( ) Yes
( ) No

74) **Webpage states that the pill is a hormonal method of contraception but contains progestin only.**

Can be used by smokers over 35, women who are breastfeeding and others who cannot use estrogen-containing methods

( ) Yes
( ) No

75) **Webpage states that the implant is a safe method of contraception.**

( ) Yes
( ) No

76) **Webpage accurately describes the risks associated with using the implant.**

- Rarely women will experience issues with removing the implant
- If a women get pregnant while the implant in inserted there is a slightly increased risk of ectopic pregnancy
- Some women develop ovarian cysts, but they usually go away without treatment
- There is a chance of infection at the page of implant insertion
- There is a chance that the implant can migrate from the initial page of implantation

Risks accurately described:

( ) Yes *(skip to 77)*
( ) No

76a) Risks incorrectly described:

____
____
____
____

76b) Risks not described:

____
____
____
____

77) **Webpage accurately describes the possible side effects of implant use.**

- Can cause weight gain
- Can cause acne
- Can cause breast tenderness
- Can cause headaches
- Can cause vaginitis
- Can cause pain at the page of insertion

Possible side effects accurately described:

( ) Yes *(skip to 78)*
( ) No

77a) Side effects incorrectly described:

____
____
____
____

77b) Side effects not described:

____
____
____
____

78) **Webpage correctly describes the non-contraceptive benefits of the implant.**

- Can be used to treat certain disorders that cause heavy bleeding and menstrual pain, such as *fibroids* and *endometriosis.*

Non-contraceptive benefits accurately described:

( ) Yes *(skip to 79)*
( ) No

78a) Non-contraceptive benefits incorrectly described:

____
____
____
____

78b) Non-contraceptive benefits not described:

____
____
____
____

79) **Webpage clearly states that the implant does not protect against STIs.**

Alternative method, such as condoms, should be used for STI protection

( ) Yes
( ) No

80) **Webpage clearly describes the possible effects of the implant on menstrual bleeding.**

- Frequent or unpredictable bleeding, especially in first few months
- Can eventually lead to no periods and reduced cramping

( ) Yes
( ) No

<u>Contraceptive Implant: Facts</u>

81) **Webpage makes it clear that getting the implant requires an appointment with healthcare provider, generally with an exam to start the method.**

( ) Yes
( ) No

82) **Webpage correctly described how much the implant usually costs, clearly stating on the page being assessed (not elsewhere on the page) that many people can get the implant at low cost or free depending on their insurance and where they live.**

Implants cost $400 to $800 (excluding exam fees), should be covered by insurance (public and private) and without cost sharing but depends on state and insurance provider policies

( ) Yes
( ) No

83) **This webpage does not make any incorrect claims about the implant.**

If it makes any of the following claims, select NO below:

- Causes abortion
- Misunderstandings of the insertion and removal procedures
- Causes infertility
- Reduces sexual desire/pleasure
- Cannot be used by nulliparous/young women

Makes false claims about the implant:

( ) Yes
( ) No *(skip to 84)*

83a) False/erroneous claims made:

____
____
____
____

Medication abortion (abortion pill)

This section assesses whether the page accurately presents each of the following pieces of information regarding medication abortion. All clinical information is based on the most recent ACOG guidance (2015) for patients on induced abortion, presenting the information considered necessary for comprehensive and accurate patient education on this topic.

<u>Medication abortion: clinical information</u>

84) **Webpage correctly identifies all of the following contraindications:**

- Confirmed or suspected ectopic pregnancy or adnexal mass
- Long-term systemic corticosteroid therapy
- Chronic adrenal failure
- Severe anemia
- Coagulopathy or anticoagulant therapy
- Current IUD use
- Uncontrolled seizure disorder
- Pregnancy greater than 10 weeks (70 days)
- Allergy to any of the medications used

Correct identification of contraindications:

( ) Yes *(skip to 85)*
( ) No

84a) Contraindications incorrectly identified:

____
____
____
____

84b) Contraindications not identified:

____
____
____
____

85) **Webpage correctly describes the procedure for receiving a medication abortion.**

A first-trimester medical abortion does not require surgery or anesthesia, but multiple visits to the health care provider are needed. Some drugs that induce abortion are taken by mouth, whereas others are inserted into the vagina. Sometimes the drugs can be taken at home. Generally, people are given oral mifepristone (Mifeprex) in clinic and oral misoprostol (Cytotec) to take at home 24-48 hours later, which approved by the FDA for use up to 70 days (10 weeks) since the first day of the last menstrual period for pregnancy termination.

Second-trimester medical abortion usually is done in a hospital or clinic where you can be monitored throughout the procedure. The medications (generally misoprostol, sometimes with mifepristone) used to cause the abortion may be put in your vagina, taken by mouth, injected into your uterus, or given through an intravenous (IV) line. These drugs cause the uterus to contract and expel the fetus. Drugs to relieve pain usually are given. Regional anesthesia often is an option. The drugs usually cause the abortion to begin within 12 hours. The abortion usually is complete within 12–24 hours, although the timing can be unpredictable. The drugs may cause side effects such

as nausea, fever, vomiting, and diarrhea. Medications to manage these side effects can be given as needed.

You will need to see your health care provider within 2 weeks to make sure the abortion is complete. If you are still pregnant after taking the medication, you may be given another dose of medication or you may need to have a surgical abortion. Continuing the pregnancy is not recommended because the drugs used for medical abortion are known to cause severe birth defects.

Procedure correctly described:

( ) Yes
( ) No

86) **Webpage correctly describes the usual patient experience during medication abortion:**

The drugs used in a medical abortion will cause bleeding that is much heavier than a menstrual period. There may be severe cramping. Nausea, vomiting, fever, and chills may occur. You can take over-the-counter pain medication. Your health care provider also may prescribe stronger pain medication if needed. It can take several days or weeks for the abortion to be complete.

( ) Yes
( ) No

87) **Webpage correctly describes possible complications of medication abortion and low risk posed by the procedure.**

Abortion is a low-risk procedure. Major complications that require hospitalization are rare. The risk of death from abortion is lower than 1 in 100,000 but increases slightly with every week of pregnancy. The risk of dying from giving birth is 14 times greater than the risk of dying from an early abortion. But as with any medical procedure, problems sometimes can occur. These can include the following:

- Incomplete abortion—If the abortion is incomplete, a follow-up procedure may be needed. This is more likely to happen with a medical abortion.
- Infection—Your health care provider will prescribe antibiotics to prevent this. Antibiotics also can be used to treat an infection if one occurs.
- Heavy bleeding—Some bleeding after an abortion is normal. Bleeding is rarely heavy enough to require a blood transfusion.

Potential complications accurately described:

( ) Yes *(skip to 88)*
( ) No

87a) Complications incorrectly described:

____
____
____
____

87b) Complications not described:

____
____
____
____

88) **Webpage correctly describes efficacy of medication abortion.**

Medication abortion has been shown to effectively terminate pregnancies 92% of the time in women with gestations up to 49 days, 92-96% in women with gestations up to 42 days, 91-95% in women with gestations from 43-49 days, and less than 85% in women with gestations beyond 49 days. Claims regarding abortion "reversal" treatment are not based on science and do not meet clinical standards

Efficacy accurately described:

( ) Yes
( ) No

89) **Webpage clearly states that medication abortions have no long-term health effects.**

Some women worry that having an abortion could affect their future health. Most health care providers agree that one abortion does not affect your ability to get pregnant or the risk of future pregnancy complications. Recent studies have shown no link between abortion and breast cancer. For women with an unplanned pregnancy, there is no difference in the risk of depression or other mental health problems between those who have an abortion and those who have the baby.

( ) Yes
( ) No

<u>Medication abortion: facts</u>

90) **Webpage makes some reference to how common abortion is in the US.**

1 in 4 women in the US will have an abortion by age 45, in 2014 14.6 women per 1,000 between 15-44 had an abortion

( ) Yes
( ) No

91) **Webpage describes the cost of medication abortion, reflecting this range of costs:**

The median price of a first trimester abortion was $490, with range of $225 to $750 and mean of $497. Women with first trimester abortions paid a median (mean) of $10 ($23) out of pocket travel costs. Cost also depends heavily on insurance coverage and state of residence.

( ) Yes
( ) No

92) **Webpage notes that some states have laws that restrict and regulate abortion, does not have to explain in detail but should mention these state laws and their potential impacts on abortion access.**

These state laws include physical and hospital requirements, gestational age limits, banning of "partial birth abortion," prohibited use of public funds to cover abortion costs, restriction of abortion coverage by private insurers, allowance of health care provider refusal, state mandated counselling, mandatory waiting periods, and the requirements of parental involvement/consent for minors seeking abortion.

( ) Yes
( ) No

93) **This webpage does not make any incorrect claims about medication abortion.**

If it makes any of the following claims, select NO below:

- Leads to increased risk of breast cancer
- Leads to future infertility
- Leads to adverse mental health outcomes or "postabortion stress"
- Leads to future miscarriage
- Leads to future ectopic pregnancy
- Leads to future preterm birth
- Can be reversed if only first pill has been taken

Makes false claims about medication abortion:

( ) Yes
( ) No *(skip to 94)*

93a) False/erroneous claims made:

____
____
____
____
____

Surgical Abortion

This section assesses whether the page accurately presents each of the following pieces of information regarding surgical abortion. All clinical information is based on the most recent ACOG guidance (2015) for patients on induced abortion, presenting the information considered necessary for comprehensive and accurate patient education on this topic.

<u>Surgical Abortion: clinical information</u>

94) **Webpage correctly identifies that there are no known absolute contraindications to surgical abortion though IUDs (if ever present) should be removed before beginning the procedure.**

Correct identification of contraindications:

( ) Yes *(skip to 95)*
( ) No

94a) Contraindications incorrectly identified:

____
____
____
____

95) **Webpage correctly describes the procedure for receiving a surgical abortion.**

A speculum is inserted into your vagina to hold it open. Your cervix usually is dilated (opened) so that a suction tube can fit through it. Your cervix is dilated either at the time of the procedure or before the procedure. When it is done at the time of the procedure, a series of dilators are inserted into and withdrawn from the cervix to gradually increase the size of the opening. When it is done before the procedure, different techniques can be used. Special dilators called laminaria can be inserted into the cervix. Medications also can be taken by mouth or placed in the vagina to dilate the cervix. After the cervix is dilated, a thin, plastic tube is inserted into the uterus. It is attached to a suction or vacuum pump, which removes the pregnancy.

Procedure accurately described:

( ) Yes
( ) No

96) **Webpage accurately describes the usual patient experience during a surgical abortion.**

Patients are under local or general anesthesia for surgical abortions, so do not experience sensation with the procedure.

( ) Yes
( ) No

97) **Webpage correctly describes the usual experience following a surgical abortion.**

After the procedure, you will rest in a recovery area. You may be able to go home as soon as an hour afterward. You can expect to have cramping for 1 or 2 days afterward. Bleeding may last for up to 2 weeks. You will be provided with antibiotics which you should take to prevent infection. You will also have a follow-up appointment 2 weeks to a month following the procedure.

( ) Yes
( ) No

98) **Webpage correctly describes the possible complications of surgical abortion and the low risk of the procedure.**

Abortion is a low-risk procedure. Major complications that require hospitalization are rare. The risk of death from abortion is lower than 1 in 100,000 but increases slightly with every week of pregnancy. The risk of dying from giving birth is 14 times greater than the risk of dying from an early abortion. But as with any medical procedure, problems sometimes can occur. These can include the following:

- Incomplete abortion—If the abortion is incomplete, a follow-up procedure may be needed. This is more likely to happen with a medical abortion.
- Infection—Your health care provider will prescribe *antibiotics* to prevent this. Antibiotics also can be used to treat an infection if one occurs.
- Heavy bleeding—Some bleeding after an abortion is normal. Bleeding is rarely heavy enough to require a blood transfusion.
- Injury to the uterus and other organs—The risk of these complications occurring during a second-trimester abortion is less than 1 in 1,000. The risk increases with the length of the pregnancy.

( ) Yes *(skip to 99)*
( ) No

98a) Complications incorrectly described:

____
____
____
____

98b) Complications not described:

____
____
____
____

99) **Webpage correctly describes efficacy of surgical abortion.**

Surgical abortion has been shown to effectively terminate pregnancies more than 99% of the time (almost always).

Efficacy accurately described:

( ) Yes
( ) No

100) **Webpage clearly states that surgical abortions have no long-term health effects.**

Some women worry that having an abortion could affect their future health. Most health care providers agree that one abortion does not affect your ability to get pregnant or the risk of future pregnancy complications. Recent studies have shown no link between abortion and breast cancer. For women with an unplanned pregnancy, there is no difference in the risk of depression or other mental health problems between those who have an abortion and those who have the baby.

( ) Yes
( ) No

<u>Surgical abortion: facts</u>

101) **Webpage makes some reference how common abortion is in the US.**

Based on recent data, 1 in 4 women in the US will have an abortion by age 45, in 2014 14.6 women per 1,000 between 15-44 had an abortion

( ) Yes
( ) No

102) **Webpage correctly describes the cost of a surgical abortion, accurately representing this range of costs:**

The median price of a first trimester abortion was $490, with range of $225 to $750 and mean of $497. The median price between 14 and less than 20 weeks was $750, with a range of $490 to $1,500 and a mean of $860. The median price at or after 20 weeks was $1,750, with a range of $946 to $6,008 and a mean of $1,874.

In addition to procedure costs, women also reported paying between $0 and $2200 for out-of-pocket travel costs

(not shown), with a median (mean) of $15 ($54). Women 14 to less than 20 weeks paid $20 and at or after 20 weeks $30. Cost also depends heavily on insurance coverage and state of residence.

( ) Yes
( ) No

103) **Webpage notes that some states have laws the restrict and regulate abortion, does not have to explain in detail but should mention these state laws and their potential impacts on abortion access.**

These state laws include physical and hospital requirements, gestational age limits, banning of "partial birth abortion," prohibited use of public funds to cover abortion costs, restriction of abortion coverage by private insurers, allowance of health care provider refusal, state mandated counselling, mandatory waiting periods, and the requirements of parental involvement/consent for minors seeking abortion.

( ) Yes
( ) No

104) **This webpage does not make any incorrect claims about surgical abortion.**

If it makes any of the following claims, select NO below:

- Leads to increased risk of breast cancer
- Leads to future infertility
- Leads to adverse mental health outcomes or "postabortion stress"
- Leads to future miscarriage
- Leads to future ectopic pregnancy
- Leads to future preterm birth

Makes false claims about surgical abortion:

( ) Yes
( ) No *(skip to 105)*

104a) False/erroneous claims made:

____
____
____
____
____

Section II: User Experience of Webpage

This section assesses the overall credibility of the webpage through the presence or absence of key components of functioning as a credible resource for users. These are:

Objectivity: Is the purpose and intention of the page clear, including any bias?

Relevance: Is the information presented appropriate for the presenter and audience?

Usability: Is the page well designed and stable?

Accuracy: Is the information presented based on timely source materials and an accurate representation of the resources used?

105) **What type of webpage is it?**

( ) Health education page
( ) Blog
( ) News page
( ) Academic resource (paper, brief)
( ) Health services page (including information and clinical services)
( ) Non-profit webpage

106) **Webpage has advertisements.**

Presence of advertisements on the webpage being assessed, indicating that the page is funded via advertisements and has a marketing strategy

( ) Yes
( ) No

107) **Organization's objectives are presented.**

Organization mission and objectives clearly presented somewhere on the webpage being reviewed.

( ) Yes
( ) No

108) **Webpage content is focused on the application of knowledge to lead to desirable behavior/outcomes.**

Users are accessing these webpages to solve their health problem (e.g. choosing a method of contraception) and the content should be focused on helping them do this rather than just providing medical facts without focus on how they can be applied.

( ) Yes
( ) No

109) **Webpage has at least one visual element (video or multimedia) that conveys or supports the main message of the page.**

Videos and/or multimedia elements available on the webpage being assessed, conveying information in ways that capture the viewer's attention

( ) Yes
( ) No

110) **Webpage is mobile friendly, meaning it displays accurately and is functional on a smartphone or tablet as well as a desktop/laptop computer.**

The webpage should have the following capabilities on a smartphone or table:

- Webpage resizes to display fully on a mobile screen
- Text-based phone numbers, physical addresses, or email addresses that can trigger a call, directions, or email message from the mobile device
- Slideshows or image rotators can function without Flash support to work with devices that do not support Flash (Apple products)
- All images are able to load quickly over mobile networks

( ) Yes
( ) No

111) **Webpage top navigation (navigation pane at the top of the page) is easy to understand and easy to use.**
Headings are concise and indicative of sub-content, subpages are consistently shown or not for all landing pages, and there is indication of users current location within the page (location indicators).

( ) Yes
( ) No

112) **Information about the googled topic can be easily found on the webpage with clear wayfinding (in headings, subheadings, etc.).**

( ) Yes
( ) No

113) **There is an internal search engine present.**

Search capability (search bar, search dropdown, etc.) for the webpage present

( ) Yes
( ) No

114) **There is a help or chat function present on the webpage, which includes having a call-in or information hotline number.**

Access to assistance with queries within the page, must be easily accessed on specific webpage being assessed either in the top or bottom link panels

( ) Yes
( ) No

115) **Webpage is written at 6-7 grade reading level.**

Page uses language understandable to the general public, meeting NIH guidelines that health materials be written at a 6-7 grade reading level based on assessment with SMOG and Flesch reading ease reading level formulas carried out on an appropriate sample of page text at http://readabilityformulas.com/

( ) Yes
( ) No

116) **Vocabulary is well explained with complicated or jargon words or ideas are used, can be defined or linked to another resource for explanation.**

Common and explicit words are used, and when complex or jargon words or ideas are used, they are easily explained, either with a clear definition in text or via a link out to provide users with more information and better understanding. **This includes links to other pages within the page and links out**

( ) Yes
( ) No

End of Quality Assessment Tool
