# Supplementary material for: Quality of top webpages providing abortion pill information for Google searches in the USA: An evidence-based webpage quality assessment
Source: PLoS One. 2021 Jan 21;16(1):e0240664. doi: 10.1371/journal.pone.0240664 (PMC7819599; doi:10.1371/journal.pone.0240664)
Supplement: S1 Table — (PDF) [file pone.0240664.s002.pdf]

S1 Table: Abortion Pill Top Webpages Full FPWQAT Scoring Results

**Assessment parameter:**	https://www.plannedparenthood.org/learn/abortion/the-abortion-pill	https://en.wikipedia.org/wiki/Medical_abortion	http://americanpregnancy.org/unplanned-pregnancy/abortion-pill/	http://www.abortionpillreversal.com/	https://www.abortionprocedures.com/abortion-pill/
**<u>User Experience of Webpage</u>**					
Type of webpage	Healthcare facility page	Non-profit page	Health education page	Health services page	Health education page
Webpage does not have advertisements.	1	1	0	1	1
Organization's objectives are presented. Organization mission and objectives clearly presented somewhere on the webpage being reviewed.	1	1	1	0	0
Webpage content is focused on the application of knowledge by users to lead to their desired behavior/outcomes.	1	0	1	0	0
Webpage has at least one visual element (video or multimedia) that conveys or supports the main message of the page.	1	0	0	0	1
Webpage is mobile friendly, meaning it displays accurately and is functional on a smartphone or tablet as well as a desktop/laptop computer.	1	1	1	0	0
Webpage top navigation is easy to understand and use.	1	1	1	1	0
Information about the googled topic can be easily found on the webpage with clear wayfinding (in headings, subheadings, etc.).	1	1	1	1	1
There is an internal search engine present.	1	1	1	0	0
There is a help or chat function present on the webpage.	1	0	1	1	1
Webpage is written at 6-7 grade reading level.	0	0	0	0	0
Vocabulary is user friendly or well explained.	1	1	0	0	0
**<u>Accuracy of Information for Medication Abortion</u>**					
Clinical Information					
Webpage correctly identifies all of the contraindications for medication abortion as outlined by ACOG	0	0	0	0	0
Webpage correctly describes the procedure for receiving a medication abortion	1	0	0	0	0
Webpage correctly describes the usual patient experience during medication abortion	1	1	1	0	0

Webpage correctly describes possible complications of medication abortion and low risk posed by the procedure	1	0	0	0	0
Webpage correctly describes efficacy of medication abortion	1	0	0	0	0
Webpage clearly states that medication abortions have no long-term health effects	1	0	0	0	0
Abortion Facts					
Webpage makes some reference to how common abortion is in the US	0	0	0	0	0
Webpage correctly describes the cost of medication abortion, reflecting a range of costs	1	1	1	0	0
Webpage explains that some states have laws that restrict and regulate abortion, does not have to explain in detail but should mention these state laws and their potential impacts on abortion access	1	0	1	0	0
Webpage does not make any incorrect claims or present any misinformation about medication abortion based on clinical evidence and current best practice for abortion providers	1	1	0	0	0
**User Experience Score (out of 11)**	**10**	**7**	**7**	**4**	**4**
**Quality of Information Score (out of 10)**	**8**	**3**	**3**	**0**	**0**
Clinical Information Score (out of 6)	5	1	1	0	0
Facts Score (out of 4)	3	2	2	0	0
**Total Score**	**18**	**10**	**10**	**4**	**4**
**Percent Achieved (out of 100%)**	**81.0%**	**42.9%**	**42.9%**	**14.3%**	**19.0%**
